# State of play and clinical prospects of antibody gene transfer

**DOI:** 10.1186/s12967-017-1234-4

**Published:** 2017-06-07

**Authors:** Kevin Hollevoet, Paul J. Declerck

**Affiliations:** Laboratory for Therapeutic and Diagnostic Antibodies, Department of Pharmaceutical and Pharmacological Sciences, KU Leuven - University of Leuven, Campus Gasthuisberg O&N 2, P.B. 820, Herestraat 49, 3000 Leuven, Belgium

## Abstract

Recombinant monoclonal antibodies (mAbs) are one of today’s most successful therapeutic classes in inflammatory diseases and oncology. A wider accessibility and implementation, however, is hampered by the high product cost and prolonged need for frequent administration. The surge in more effective mAb combination therapies further adds to the costs and risk of toxicity. To address these issues, antibody gene transfer seeks to administer to patients the mAb-encoding nucleotide sequence, rather than the mAb protein. This allows the body to produce its own medicine in a cost- and labor-effective manner, for a prolonged period of time. Expressed mAbs can be secreted systemically or locally, depending on the production site. The current review outlines the state of play and clinical prospects of antibody gene transfer, thereby highlighting recent innovations, opportunities and remaining hurdles. Different expression platforms and a multitude of administration sites have been pursued. Viral vector-mediated mAb expression thereby made the most significant strides. Therapeutic proof of concept has been demonstrated in mice and non-human primates, and intramuscular vectored mAb therapy is under clinical evaluation. However, viral vectors face limitations, particularly in terms of immunogenicity. In recent years, naked DNA has gained ground as an alternative. Attained serum mAb titers in mice, however, remain far below those obtained with viral vectors, and robust pharmacokinetic data in larger animals is limited. The broad translatability of DNA-based antibody therapy remains uncertain, despite ongoing evaluation in patients. RNA presents another emerging platform for antibody gene transfer. Early reports in mice show that mRNA may be able to rival with viral vectors in terms of generated serum mAb titers, although expression appears more short-lived. Overall, substantial progress has been made in the clinical translation of antibody gene transfer. While challenges persist, clinical prospects are amplified by ongoing innovations and the versatility of antibody gene transfer. Clinical introduction can be expedited by selecting the platform approach currently best suited for the mAb or disease of interest. Innovations in expression platform, administration and antibody technology are expected to further improve overall safety and efficacy, and unlock the vast clinical potential of antibody gene transfer.

## Purpose of the review

This review provides an elaborate overview of the state of play and clinical prospects of in vivo antibody gene transfer. Focus includes hallmarks of the applied expression platforms, key pre-clinical and clinical studies, recent innovations, opportunities and remaining clinical hurdles.

## Recombinant therapeutic antibodies

### Therapeutic market and impact

In 1986, the clinical approval of the first monoclonal antibody (mAb), Orthoclone OKT3, initiated a new era in biological therapeutics. Since then, mAb products have grown to become the dominant class within the biopharmaceutical market [1, 2]. mAbs today are approved for the treatment of cancer and autoimmune, inflammatory and infectious diseases [3–5]. Applications thereby range from a few thousand patients or less for orphan indications to millions of patients for diseases such as asthma and rheumatoid arthritis [1]. A variety of mAb products have been established, ranging from conventional full-length immunoglobulins, mostly isotype G (IgG), to fusion proteins and minimal fragments. As of May 2017, 63 mAb products have been approved in the US or Europe for therapeutic use [6]. In 2013, 18 mAb products achieved annual sales of over $1 billion, with six of them (adalimumab, infliximab, etanercept, rituximab, bevacizumab and trastuzumab) having sales of more than $6 billion [1]. In addition, immune checkpoint inhibiting mAbs have recently reignited the field of cancer immunotherapy. This market segment alone is expected to increase from approximately $1 billion in 2013 to in excess of $7 billion in 2020 [7]. At the current approval rate of approximately four new products per year, about 70 mAb products will be on the market by 2020, with a projected combined world-wide sales of nearly $125 billion [1]. As the biopharmaceutical industry further evolves, the number and types of diseases that can benefit from mAb products will continue to increase [2].

### Current issues

#### Production cost and product pricing

Price tags of $100,000 or more per mAb treatment course are no longer an exception [8, 9]. The large size and complex nature of mAb biologics require a costly production and purification process, and extensive downstream quality control. Manufacturing of mAbs is therefore far more expensive than e.g. small molecules or antibiotics. This clearly impacts the cost, but it is not the main contributor to the final product price. With economies of scale into play, production costs are around $50–100 per gram of mAb [10]. In contrast, US wholesale prices in the first quarter of 2015, e.g. in the field of immune checkpoint inhibitors, ranged between $29,000 and $157,000 per gram of mAb [8]. Thus, the price point set by early innovative treatments plays an important role, while expenses related to research and development, clinical trials, royalties, failed products, and marketing further add to the overall price [9, 11].

#### Parenteral administration

Depending on the disease indication and stage of treatment, patients can require high-dose mAb administration as frequent as every 2 weeks for a prolonged period of time. The majority of approved mAbs are administered by intravenous (i.v.) infusion. Drawbacks of this delivery route are the fluctuating mAb pharmacokinetics (peaks and troughs), risk of bloodstream infections, hours-long administration, need for a hospital setting, and infusion-related adverse events [12, 13]. Subcutaneous (s.c.) injection is rapidly gaining ground as a more practical alternative. It is generally limited to a few minutes, may eventually be suited for self-administration at home, and results in less fluctuating mAb pharmacokinetics [14]. Because the volume of injection has to be limited (1–5 ml) for pain reasons, s.c. formulation may require excipients that facilitate administration. In addition, the product needs to diffuse in the extracellular matrix to reach the blood, resulting in a delay in absorption and lower bioavailability compared to i.v. injection. Regarding specific side-effects, s.c. delivery can lead to injection-site reactions, including erythema and pain, and may be more immunogenic than i.v. administration [14]. Irrespective of the route of administration, systemic mAb circulation can evoke problems. These include dismal efficacy due to difficulties in reaching the target, e.g. penetrating solid tumors [15, 16] or crossing the blood–brain barrier [17], or specific systemic side-effects, e.g. with immune checkpoint inhibitors such as ipilimumab [18, 19], a mAb targeted at cytotoxic T-lymphocyte associated protein 4 (CTLA-4). More local administration routes, e.g. the tumor, are pursued, but most are in an early clinical phase.

### Recap

The issues with regard to the cost and dosing of conventional mAb therapy can restrict (i) access to therapy, (ii) implementation of more effective treatment modalities, e.g. mAb combinations [3, 8], and (iii) penetration into cost-sensitive indications or markets, including infectious diseases and prophylactic use [20]. Overall, these hurdles clearly illustrate the need for innovations in mAb production and administration.

## The case for antibody gene transfer

### Concept

**Fig. 1:**
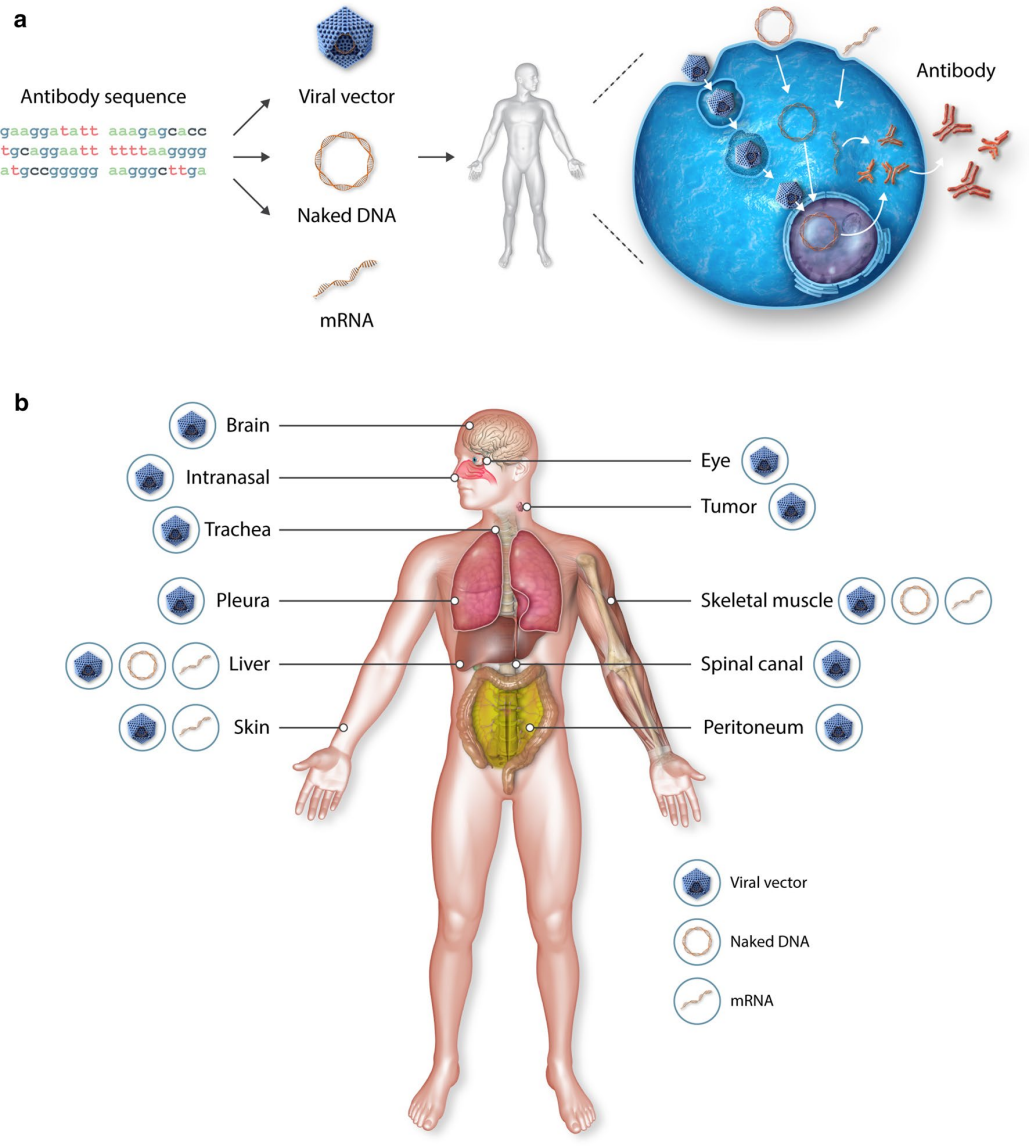
Principle and versatility of antibody gene transfer. **a** Schematic overview of the basic principle of antibody gene transfer. Starting from the antibody sequence, the encoding nucleotides are placed into viral vectors (adenovirus, adeno-associated virus, or oncolytic virus), naked DNA (plasmid or minicircle), or messenger RNA (mRNA), and administered to the host. Following injection, the encoding nucleotides enter the cells after which antibody production can commence. **b** Sites in the body potentially amendable to clinical antibody gene transfer administration or production, based on pre-clinical and clinical antibody gene transfer studies with the three different expression platforms. The muscle and liver (via intravenous delivery) have been most often reported. Others include the brain [34, 37, 75–78], eye [81], intranasal route [38, 55, 72, 79, 80], trachea [56], tumors (either directly injected or via intravenous delivery [30, 36, 39, 99–101, 103, 104, 106–109]), pleura [57, 82, 83], peritoneum [45, 60, 84], skin (intradermal [44] and subcutaneous [45]), and spinal canal [40]

In vivo antibody gene transfer seeks to administer to patients the mAb-encoding nucleotide sequence, rather than the mAb protein. This allows the patient’s body to produce the therapeutic mAb of interest for a prolonged period of time, and secrete it either systemically or locally, depending on the production site (Fig. 1). Gene-based antibody therapy presents a labor- and cost-effective alternative to the conventional production, purification and administration of mAb proteins. Three antibody expression platforms have been pursued in vivo: viral vectors, naked DNA and RNA (Fig. 1a), each of which are cheaper to produce than mAb proteins. Antibody gene transfer can enable cost-savings by reducing the cost of goods and of production, and the frequency of drug administration. Overall, a prolonged in vivo production of mAbs can contribute to (i) a broader therapeutic or prophylactic application of mAbs in price-sensitive conditions, (ii) an improved accessibility to therapy in both developed and developing countries, and (iii) more effective and affordable treatment modalities, e.g. by facilitating nucleotide-based mAb cocktails or local mAb expression. In addition to in vivo antibody gene transfer, cells can be harvested from the host, engineered to produce mAbs and re-administered (reviewed in [21, 22]). This ex vivo antibody gene transfer is beyond the scope of the current review.

### Applications

**Fig. 2:**
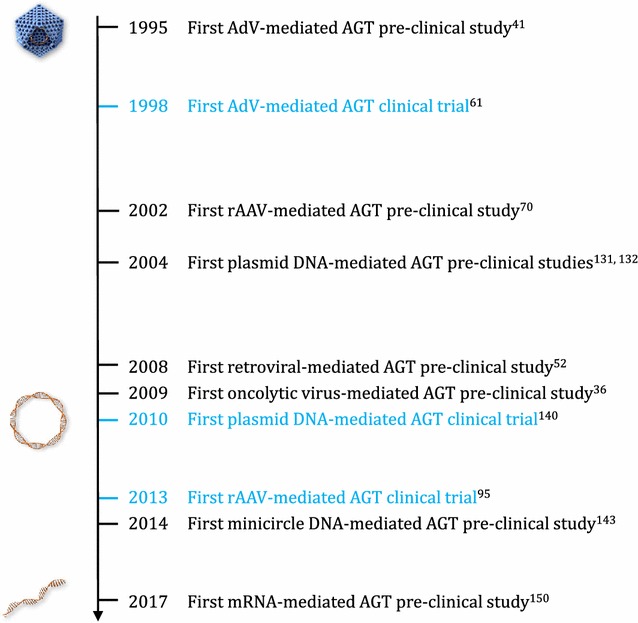
Timeline of antibody gene transfer milestones. The first peer-reviewed pre-clinical studies or clinical trials for each of the different expression platforms (viral vectors, naked DNA, and mRNA) are ranked in chronological order. *AdV* adenovirus, *rAAV* recombinant adeno-associated virus, *AGT* antibody gene transfer, *mRNA* messenger RNA. Illustrations from *top* to *bottom* represent the viral, naked DNA and mRNA expression platforms

The history of pre-clinical and clinical studies of antibody gene transfer spans more than two decades (Fig. 2), and reflects the continuous innovations in the applied expression platforms. In line with the broad applicability of mAbs, antibody gene transfer has been used in a myriad of indications including cancer, infectious diseases, inflammatory diseases and central nervous system (CNS) diseases (Table 1). In addition to full-length IgG, in vivo expressed mAb products include antibody-protein fusion products (e.g. immunoadhesins [23, 24]), bispecifics [25–27] and fragments (e.g. antigen-binding fragment (Fab) [28–30], single-chain variable fragment (scFv) [31–41], and single-domain antibodies [27, 42–45]). Figure 1b depicts the sites on the body potentially amendable to antibody gene transfer, based on pre-clinical and clinical studies. Intramuscular antibody gene administration has been most widely evaluated (reviewed in [46]), and also carries the highest clinical translatability and application. Indeed, the inherent anatomical, cellular and physiological properties of skeletal muscle make it a stable environment for long-term mAb expression and systemic circulation [47]. Skeletal muscle is easily accessible, allowing multiple or repeated administrations. The abundant blood vascular supply provides an efficient transport system for secreted mAbs into the circulation. The syncytial nature of muscle fibers allows dispersal of nucleotides from a limited site of penetration to a large number of neighboring nuclei within the fiber. Skeletal muscle fibers are also terminally differentiated cells, and nuclei within the fibers are post-mitotic [47, 48]. As a consequence, integration in the host genome is not a prerequisite to attain prolonged mAb expression [48]. The liver is another site often used for pre-clinical antibody gene transfer, and is typically transfected via i.v. injection. This organ has various physiological functions, including the synthesis of plasma proteins. While this makes it potentially well suited for in vivo mAb production, accessibility beyond i.v. injection presents a challenge. The tumor presents another popular site for pre-clinical antibody gene transfer, targeted either via i.v. or direct injection in pre-clinical studies. It carries high clinical relevance, despite lacking the accessibility, stability and homogeneity the muscle is touted for. Indeed, intratumoral mAb expression can allow for a local production of the therapeutic, waiving the need for high systemic mAb levels often required to penetrate and impact solid tumors [15, 16]. A similar reasoning applies for the brain, which is frequently targeted in the context of antibody gene transfer to avoid the difficulties with blood–brain barrier trafficking [17].

**Table 1:** Pre-clinical antibody gene transfer studies ranked according to expression platform and indication

Expression platform	Disease indication		References
Viral vector			
AdV	Cancer		[31, 32, 59]
	Infectious diseases	*Clostridium botulinum*	[45]
		*Clostridium difficile*	[43]
		Influenza	[55]
		Respiratory syncytial virus	[57]
		*Yersinia pestis*	[38, 166]
		West Nile virus	[167]
	Other	Pulmonary edema	[56]
rAAV	Cancer		[71, 83, 84, 168–172]
	CNS	Alzheimer’s disease	[35, 37, 73–78, 173, 174]
		Huntington disease	[76, 173]
		Prion disease	[33, 34, 175]
	Infectious diseases	Anthrax	[42, 82]
		Ebola	[72]
		Hepatitis C	[176]
		HIV (in mouse)	[70, 177–179]
		SIV (in rhesus macaque)	[23, 24, 90–92]
		Influenza	[55, 79, 80, 180]
		Malaria	[181]
		Respiratory syncytial virus	[57]
	Other	Addiction	[182, 183]
		Gelsolin amyloidosis	[27]
		Contraception	[184]
		Macular degeneration	[81]
Oncolytic virus	Cancer		[30, 36, 39, 99–101, 103, 104, 106–109]
Naked DNA			
pDNA	Auto-immune disease		[131]
	Cancer		[141, 185, 186]
	Infectious diseases	Chikungunya virus	[29]
		Dengue virus	[136]
		Ebola	[137, 138, 187]
		Hepatitis B	[133]
		HIV	[28]
		Influenza	[134]
		Pneumonia	[25]
mcDNA	Cancer		[26]
	Inflammatory diseases	Arthritis	[143]
		Skin allograft survival	[144]
RNA			
mRNA-LNP	Cancer		[44]
	Infectious diseases	*Clostridium botulinum*	[44]
		HIV	[150]
		Influenza B	[44]
		Rabies	[44]

*AdV* adenovirus, *CNS *central nervous system, *rAAV* recombinant adeno-associated virus, *pDNA* plasmid DNA, *mcDNA* minicircle DNA, *HIV* human immunodeficiency virus, *SIV* simian immunodeficiency virus, *mRNA-LNP* messenger RNA encapsulated in lipid nanoparticles

## Viral vector-mediated antibody gene transfer

### Rationale

Viral vectors are currently used as a delivery vehicle in the vast majority of pre-clinical and clinical gene therapy trials [49]. The main driver thereto is their exceptional gene delivery efficiency, which reflects a natural evolutionary development. Vector drawbacks include a complex production, a limited packaging capacity for incorporation of exogenous DNA, immunogenicity, cytotoxicity, and, in some cases, risk of insertional mutagenesis [50, 51]. Adenoviruses (AdV) and adeno-associated viruses (AAV) are most often applied for gene therapy applications [49], including pre-clinical antibody gene transfer. Retroviruses have been used in only a very limited number of antibody gene transfer studies [52, 53], which is likely related to their inherent risk of insertional mutagenesis. These reports are not elaborated on in the current review.

### Adenoviral vectors

AdVs are non-enveloped, double-stranded DNA viruses that neither integrate in the host genome nor replicate during cell division [54]. As early as 1995, Deshane et al. [41] reported on the intraperitoneal delivery of an AdV-based vector to express an anti-human epidermal growth factor receptor 2 (HER2) scFv intrabody in mouse cancer models. In subsequent years, AdV-mediated antibody gene transfer has shown therapeutic efficacy in different pre-clinical disease models (Table 1). Systemic mAb expression has mostly been pursued, via s.c. [45] and especially i.v. and intramuscular AdV injection (reviewed in [46]). A series of studies has focused on a more local mAb production in mice, either via intranasal [38, 55], intratracheal [56] or intrapleural administration [56, 57] of the encoding AdV (Fig. 1b). The use of AdVs as oncolytic vectors is discussed in a separate section. Overall, AdV-mediated mAb expression has shown to be highly variable and fairly transient (reviewed in [22]). Peak serum concentrations higher than 1 mg/ml have been reported a few days after AdV delivery. Within 1 week mAb titers typically began to decline, and long-term concentrations ranging from 20 ng/ml to 40 µg/ml have been reported [58, 59].

Building on their earlier pre-clinical work [41, 60], Alvarez et al. initiated in 1998 a Phase I trial to evaluate a single intraperitoneal administration of an AdV dose coding for an anti-HER2 scFv intrabody [61, 62]. Fifteen patients with recurrent ovarian HER2+ cancer were included. No dose-limiting vector-related toxicity was reported. In ascites, intrabody expression was detected in 11 of 14 evaluable patients 2 days after AdV administration and in eight out of 13 evaluable patients on day 56. In cell pellets from the ascites, intrabody expression was present in ten of 14 evaluable patients at day 2, a number that decreased to five out of 12 evaluable patients at day 14. By day 56, only one out of 11 evaluable patients still had detectable intracellular expression. All study patients had detectable serum antibodies to AdV prior to treatment. Serial serum samples were obtained up to day 56 in six patients. All but one of the six patients had an increase in anti-AdV antibody titers [62]. No follow-up clinical studies of this particular trial have been reported. In line with the study findings, many AdVs are indeed highly prevalent in the general population [63], and pre-existing immunity can limit the clinical efficacy of AdV-mediated gene transfer. Of note, the first gene therapy death in 1999 was a direct consequence of inflammatory immune responses and toxicity against an AdV [64], illustrating the safety issues linked to vector immunogenicity. Overall, the prevalence of pre-existing anti-AdV immunity coupled with the transient nature of the resulting mAb expression has limited enthusiasm for AdVs [22].

### Adeno-associated viral vectors

AAVs are non-enveloped small, single-stranded DNA viruses capable of infecting both dividing and non-dividing cells. Similar to AdV, AAV-based vectors remain in an episomal state in the nucleus and display a limited risk of integration [65, 66]. In contrast to the limited durability of AdV-mediated gene transfer, transgene expression can persist for years following intramuscular recombinant AAV (rAAV) vector delivery [67].

Alipogene tiparvovec (Glybera™), an rAAV encoding the human lipoprotein lipase gene, was approved in 2012 as the first gene therapy product in Europe [68]. Market authorization, however, did not translate into commercial success. The product received intense scrutiny for its $1 M price tag [69] and failed to penetrate its niche market. In April 2017, the company announced that it will not pursue renewal of the marketing authorization in Europe when it is scheduled to expire in October 2017. This decision was not related to any efficacy or safety issue, but merely driven by its very limited use. Indeed, various rAAV-based gene therapy products are currently under clinical evaluation.

In the context of antibody gene transfer, Lewis et al. [70] in 2002 were the first to demonstrate in vivo production of an anti-human immune deficiency virus (HIV) mAb in mice following intramuscular injection of the mAb-encoding rAAV. Although relatively low-level mAb production was observed in vivo (<10 µg/ml), expression persisted for at least 6 months, and a clear dose–response was observed between the amounts of administered vector and resulting mAb titers [70]. Further improvements in expression cassette design have led to peak serum mAb levels in the single-digit mg/ml level in mice, with sustained production up to 1 mg/ml for months following rAAV delivery [71]. Similar results have been reported since (reviewed in [22, 46]), and rAAV-mediated antibody gene transfer has shown efficacy in a myriad of pre-clinical disease models (Table 1). Its potential for combination therapy has also been demonstrated, i.e. by expressing two mAb components of the anti-Ebola ZMapp™ [72]. Similar to AdV, intramuscular and i.v. rAAV administration have been most often pursued (reviewed in [46]). A variety of additional delivery sites have been probed to achieve a more local therapeutic effect. These include the intracranial [34, 37, 73–78], intranasal [72, 79, 80], intravitreal [81], intrathecal [40], intrapleural [82, 83], and intraperitoneal route [84] (Fig. 1b).

rAAV-mediated antibody gene transfer has made most progress in the field of human immunodeficiency virus (HIV) (reviewed in [46, 85–88]), a relevant disease indication. Indeed, current HIV vaccines fail to generate neutralizing antibodies that prevent HIV infection and acquired immune deficiency syndrome (AIDS). The last 5–10 years has seen an accumulation of potent, broadly-neutralizing mAbs (bnAbs) against HIV [89]. However, the cost and frequent infusion associated with conventional mAb administration hampers their therapeutic or prophylactic application, paving the way for alternatives such as antibody gene transfer. Several antibody gene transfer studies in rhesus monkeys, weighing 2–17 kg, against simian immunodeficiency virus (SIV) have been conducted [23, 24, 90–92]. In an initial study by Johnson et al. [23] in 2009, rhesus macaques received intramuscular injection of rAAVs coding for various anti-SIV immunoadhesins (antibody-protein fusion molecules). Six of the nine monkeys receiving rAAV-based immunoadhesins were protected after SIV challenge, while all six naïve controls became infected. The three monkeys from the rAAV-immunoadhesin group that became infected had developed a humoral antibody immune response to the immunoadhesins, leading to undetectable immunoadhesin levels 4 weeks after administration, the time of SIV challenge. In the protected animals, immunoadhesin titers ranged between 3 and 190 µg/ml at the time of SIV challenge, depending on the type of rAAV used. Immunoadhesin titers peaked around 6 months after rAAV injection, reaching 400 μg/ml in some animals [23]. Longitudinal studies of the protected monkeys, more than 6 years post-injection, showed that immunoadhesin levels dropped after 2 years to a stable level of approximately 20 μg/ml, which was maintained for at least 4 years [93]. A subsequent study converted some of these immunoadhesins into authentic IgG, resulting in anti-SIV mAbs that contained only rhesus IgG sequences [94]. rAAV-mediated delivery, however, was unable to obviate a humoral response against the expressed mAbs [91]. In a follow-up study, the magnitude of the anti-antibody responses was shown to correlate with the sequence divergence of the delivered mAb from the germline, even in fully rhesus mAbs [92]. Saunders et al. [90] also experienced the restrictive nature of antibody-mediated immunity when expressing a “rhesusized” mAb. Only when the host immune system was suppressed with cyclosporine A, the rAAV-expressed rhesusized mAb could circulate in macaques for 16 weeks at serum levels up to 66 μg/ml [90]. Finally, Gardner et al. [24] injected rhesus macaques intramuscularly with an rAAV encoding the anti-HIV rhesus eCD4-Ig, a fusion protein based on the immunoadhesin CD4-Ig. As a result, 17–77 μg/ml of eCD4-Ig was expressed for more than 40 weeks in circulation, and macaques were protected from several SIV challenges. Two of four monkeys had a weak anti-eCD4-Ig response, the other two showed none. Of note, rAAV-expressed rhesus forms of bnAbs elicited higher anti-antibody responses compared to the rhesus eCD4-Ig [24]. This could relate to the extensive sequence identity with germline sequences and the minimal non-germline sequences of eCD4-Ig [92]. In 2013, the International AIDS Vaccine Initiative initiated the first Phase I clinical trial of rAAV-mediated antibody gene transfer to evaluate safety and tolerability of intramuscular injection of rAAV-encoding PG9, an HIV-bnAb [95]. As of May 2017, no interim results have been reported, and recruitment reportedly is still ongoing (ClinicalTrials.gov: NCT01937455). With robust data in rhesus macaques and an ongoing clinical trial, rAAV is currently the platform of choice for intramuscular viral-vectored antibody gene transfer.

### Oncolytic viruses

Oncolytic viruses promote anti-tumor responses through selective tumor cell killing and induction of systemic anti-tumor immunity [96]. The mechanisms of action are not fully elucidated but are likely to depend on viral replication within transformed cells, induction of primary cell death, interaction with tumor cell anti-viral elements and initiation of innate and adaptive anti-tumor immunity [96]. Many of the oncolytic viruses that are currently in the clinic have a natural tropism for cell surface proteins that are aberrantly expressed by cancer cells. To date, AdV, poxviruses, coxsackieviruses, poliovirus, measles virus, Newcastle disease virus, reovirus, and others have entered into early‐phase clinical trials [96]. In 2015, the FDA and EMA approved talimogene laherparepvec (T-VEC, Imlygic™), an oncolytic herpes virus armed with the gene for granulocyte–macrophage colony-stimulating factor (GM-CSF) [96, 97]. The self-perpetuating nature of oncolytic viruses makes them an appealing platform for antibody gene transfer, as transgene products can be amplified along with viral replication, thereby maximizing therapeutic effect [98].

The first category of mAbs used to arm oncolytic viruses were the tumor-targeting mAbs. Local intratumoral expression presents an appealing strategy to overcome poor mAb penetration in solid tumors [15, 16]. In a first, Frentzen et al. [36] in 2009 armed replication-competent oncolytic vaccinia viruses with a scFv directed against both human and murine vascular endothelial growth factor (VEGF). Following i.v. injection, tumor-specific delivery and continued scFv production was obtained in mouse human lung cancer xenograft models. Serum scFv levels were detected up to 37 days after virus injection, with peak levels of 1.4 µg/ml. Corresponding scFv levels in tumor fluid were 12–15 times higher. The anti-VEGF-scFv armed virus had a better anti-tumor response than the unarmed virus. The enhanced efficacy was comparable to treatment of tumors with a one-time i.v. injection of the unarmed vector and concomitant multiple intraperitoneal injections of the anti-VEGF bevacizumab [36]. Building on these results, the same group applied this principle in several mouse human cancer models [39, 99, 100] and in mouse canine xenograft models [101, 102], paving the way towards veterinary medicine. Combination therapy was also pursued. Following i.v. administration, armed vaccinia viruses induced a constitutive intratumoral expression of scFvs against VEGF, epidermal growth factor receptor, and fibroblast activation protein [103]. Another group recently reported similar findings following intratumoral injection of an oncolytic AdV armed with full-length anti-HER2 trastuzumab [104].

Immunomodulatory mAbs present another, potentially more relevant category to arm oncolytic viruses. Indeed, for oncolytic virus therapy, it is desirable to override immune checkpoint inhibitor networks and thereby create a pro-inflammatory environment within the cancer. Numerous Phase I trials are currently underway to evaluate the combination of oncolytic viruses and conventional immunomodulatory mAb administration [96, 105]. However, systemic treatment with checkpoint-blocking mAbs can lead to severe immune-related adverse effects [18, 19], highlighting the opportunity for local therapies, e.g. via mAb-armed oncolytic viruses. Different studies have pursued this approach in mouse cancer models. Dias et al. [106] in 2012 armed a replication-deficient and -competent oncolytic AdV with an anti-human CTLA-4 mAb. Following intratumoral delivery in nude mice xenograft models, the armed replication-competent virus demonstrated an improved anti-tumor effect compared to the unarmed virus, despite the lack of immunological function the anti-human CTLA-4 mAb had in these mice [106]. A week after intratumoral injection of the armed replication-competent oncolytic virus, mAb levels in tumors and plasma were 17 and 0.4 mg/ml, respectively. Levels were significantly higher compared to those obtained with the replication-deficient armed virus, but no significant difference in tumor response was observed [106]. In another study, i.v. injection of a replicating AdV expressing an anti-murine CTLA-4 mAb delayed tumor growth in syngeneic mouse models, and led to complete regressions when combined with a virus encoding GM-CSF. Data on mAb expression was not reported [107]. Similar results were obtained with daily intratumoral injections for a period of 4–5 days of an attenuated measles virus encoding scFv-Fc fusion proteins against CTLA-4 or programmed cell death-ligand 1 (PD-L1) [108]. Another recent pre-clinical study armed oncolytic vaccinia viruses with anti-murine programmed cell death protein 1 (PD-1) Fab, scFv or full-length mAb [30]. Reflecting virus replication, mAb levels in the tumor peaked 3–5 days after intratumoral injection at 9 or 30 µg/ml, depending on the tumor model. Serum mAb levels followed the same trend, albeit threefold or more lower, although mAb detection was lost after 5 days. Intratumorally expressed mAbs lasted longer compared to intratumoral injection of anti-PD-1 mAb protein, with follow-up limited to 11 days after injection. Fab and scFv expression were not reported. Anti-tumor responses of the virus armed with either the anti-PD-1 scFv or mAb were superior to the unarmed virus and as effective as the combination of the unarmed virus and systemic anti-PD-1 mAb protein injections [30]. Most recently, intratumoral administration of a combination of an oncolytic AdV and a helper-dependent AdV, armed with an anti-PD-L1 mini-antibody (a scFv CH2-CH3 fusion protein), improved the anti-tumor effect of chimeric antigen receptor (CAR) T cell therapy in mice [109]. The benefits of locally produced anti-PD-L1 mini-antibody could not be achieved by anti-PD-L1 IgG infusion plus CAR T-cells and co-administration of an unarmed AdV [109].

Overall, these results illustrate the therapeutic potential of mAb-armed oncolytic viruses, although some questions remain. None of the above studies evaluated the occurrence of a humoral or cell-mediated response against the expressed mAb or viral vector, factors that can impact prolonged transgene expression in immune competent animals. Furthermore, while replication competence can boost mAb expression, it also carries biosafety concerns.

### Recap

Pioneered by AdV, the field of viral vector-mediated antibody gene transfer made significant strides in the past decades. The myriad of successfully evaluated administration routes, pre-clinical models and disease indications put the capabilities of antibody gene transfer at full display. rAAV and muscle emerged as respectively the vector and administration site of choice for prolonged mAb expression. In the context of vectored intratumoral antibody gene transfer, oncolytic viruses have a distinct advantage, as they can specifically target tumor cells, boost mAb expression, and amplify therapeutic responses. Moving forward, vector-mediated delivery still faces several issues that may limit its broad clinical use, with the various flavors of immunogenicity being the most critical [51, 63, 87]. First, a significant portion of the population has already been exposed to the applied viruses and thus harbors pre-existing immunity [22, 110]. The presence of pre-existing or induced antibody-based immunity against the viral vector can significantly decrease the efficacy of vectored expression strategies, and also limit the utility of the same serotype of a vector for repeated administration [87]. Second, cell-mediated response against the vector particle or transgene product can eliminate the transduced cells, whereas the innate response can cause local and/or systemic toxicity and enhance a secondary antigen-dependent immune response [111]. Third, for oncolytic viruses specifically, a functional adaptive immune system can restrain viral multiplication [96], providing a source of uncertainty in a clinical setting. Fourth, a humoral antibody response against the expressed mAb can lead to a rapid loss of mAb detection, as illustrated repeatedly in the rAAV non-human primate (NHP) studies. In response, various strategies are currently under development to evade or prevent these different immune responses [88, 112, 113].

## DNA-mediated antibody gene transfer

### Rationale

In 1990, Wolff et al. [114] showed how injection of naked plasmid DNA (pDNA) into the skeletal muscle of mice led to the local expression of the encoded protein, kick-starting the field of DNA-based therapeutics. pDNA waives the need for a virus as biological vector, and presents an appealing platform for antibody gene transfer. Compared to viral vectors, pDNA is considered low-immunogenic (allowing e.g. repeated dosing), is cheaper to produce, ship, and store, and has a much longer shelf-life. After entry in the nucleus, pDNA remains in a non-replicating non-integrating episomal state, and is lost during the breakdown of the nuclear envelope at mitosis. pDNA has no defined restrictions regarding the size of the transgene compared to viral vectors, and its modular nature allows for straightforward molecular cloning, making them easy to manipulate and design for therapeutic use [115]. Plasmids are used in about 17% of the ongoing or completed gene therapy clinical trials [49], and showed to be well-tolerated and safe [116]. A plasmid-based pharmaceutical for humans has not been marketed, although several clinical trials entered Phase II–III [49]. The main disadvantage of pDNA compared to viral vectors is the lower transgene expression, which the field continues to address by innovating DNA administration and construct design.

### DNA administration

The method of DNA administration can greatly improve transgene expression. In vivo DNA-mediated antibody gene transfer has been exclusively reported with physical methods of transfection, i.e., electroporation or hydrodynamic injection. Electroporation presents the propagation of electrical fields within tissues, which induces a transient increase in cell membrane permeability [117]. Electrotransfer of DNA is a multistep process, involving (i) electrophoretic migration of DNA towards the plasma membrane, (ii) DNA accumulation and interaction with the plasma membrane, and (iii) intracellular trafficking of the DNA to the nucleus, after which gene expression can commence [117]. The first gene electrotransfer clinical trial was initiated in 2004 [118]. By 2015, over 50 trials had used electrotransfer for direct gene delivery in vivo or ex vivo [117]. Therapeutic gene electrotransfer approaches include DNA vaccines, immunotherapeutic agents, cell-growth inhibitors, pro-apoptotic agents, tumor antigens, and anti-angiogenic agents. Intramuscular, intratumoral and intradermal administration have been evaluated in clinical trials [117, 119]. In human subjects, application of electric pulses reportedly led to short-term pain or discomfort [120]. This transient pain can vary depending on the electrical parameters, the electrode used, and site of pulse delivery. It may be diminished or ultimately eliminated, e.g. by decreasing the applied voltage or by varying pulse frequency [119]. To support consistent procedure application independent of operator skill or experience, a great deal of efforts has focused on the integration of DNA administration and electroporation application into a single automated device [119].

Hydrodynamic-based transfection implies the i.v. injection of high volumes of pDNA, driving DNA molecules out of the blood circulation and into tissue. In mice models, hydrodynamic tail injection (HTI) is typically used for liver transfection. This technique might be amenable to use in humans but would be limited to locations at which a temporary increase in pressure could be created, e.g. by using a blood-pressure cuff applied to a limb [121]. Other potentially less invasive physical delivery methods include sonoporation and magnetofection, but these currently lack application for antibody gene transfer and clinical evaluation [122]. DNA uptake can also be improved by complexing the molecules with chemical delivery vehicles (e.g. cationic lipids or polymers and lipid nanoparticles) [123]. While these methods are less invasive than physical methods, they are in the early phases of clinical development. None have been applied for in vivo DNA-mediated antibody gene transfer.

### DNA backbone

In addition to the choice of delivery method, transgene expression can be improved by modifying the make-up of pDNA constructs [115, 124]. Conventional pDNA consists of a transcription unit and bacterial backbone. The transcription unit carries the encoding sequence along with regulatory elements. The bacterial backbone includes elements like an antibiotic resistance gene, an origin of replication, unmethylated CpG motifs, and potentially cryptic expression signals. Some of these sequences are required for the production of plasmid DNA, but each of them can carry biological safety risks (reviewed in [125]). Moreover, the unmethylated bacterial CpG motifs can be immunostimulatory, resulting in silencing the expression of the encoded transgene [126–128]. For therapeutic gene therapy, including antibody gene transfer, the presence of a bacterial backbone is clearly counterproductive. Of the different types of available minimal vectors [124], so far only minicircle DNA (mcDNA) has been reported for antibody gene transfer. Minicircles are plasmid molecules devoid of bacterial sequences, generated via a process of recombination, restriction and/or purification [124]. Elimination of the bacterial backbone has shown higher transfection efficiency and prolonged transgene expression in a variety of tissues [124, 128–130]. Despite the benefits of these minimal vectors, clinical introduction is lagging behind because of the challenging manufacturing. Ongoing advances in production and purification are expected to overcome these technical hurdles and promote clinical introduction [124].

### Plasmid DNA

The first reports in mice on the use of pDNA for intramuscular antibody gene electrotransfer date back from 2004 [131, 132]. Modest serum mAb titers were attained, ranging from a few hundred nanogram to a single microgram per ml [131, 132]. Since then, proof of concept has been demonstrated in different mouse disease models, mainly in infectious diseases (Table 1). Despite renewed interest in recent years, the use of pDNA still lags behind that of viral vectors. This is in part related to the significantly lower mAb titers associated with DNA-based gene transfer. To expand its application, different approaches have pursued a higher mAb expression or overall efficacy in mice. A first strategy simply relies on giving multiple or repeated pDNA doses [133, 134]. Electrotransfer of 30 µg pDNA in three muscles each instead of one, e.g., led to mAb serum titers up to 20 µg/ml [134]. A second approach relates to the use of a delivery adjuvant. pDNA electrotransfer can be enhanced by pre-treating the muscle with hyaluronidase, an enzyme that transiently breaks down hyaluronic acid, decreasing the viscosity of the extracellular matrix and facilitating DNA diffusion [135]. For antibody gene transfer, this led to an increase in mAb expression by approximately 3.5-fold, achieving plasma peak titers of 3.5 µg/ml with 30 µg pDNA [134]. A third strategy focuses on antibody or cassette engineering. Following codon-, RNA- and leader sequence-optimization, peak serum mAb or Fab titers of 1–3 µg/ml have been attained with intramuscular electrotransfer of 25–100 µg ‘optimized’ pDNA [28, 29, 136]. However, expression titers from the ‘non-optimized’ pDNA went largely unreported, making it difficult to appreciate the impact of these modifications. Recently presented data showed how framework grafting and/or scFv-Fc conversion could increase serum levels of some poorly expressed mAbs up to a tenfold, resulting in peak titers of 6 µg/ml [137]. For mAbs that already showed high expression in an unmodified state, this engineering increased serum peak levels by about a threefold. Intramuscular electrotransfer of 200 µg pDNA thereby resulted in titers of approximately 20–30 µg/ml of the grafted scFv-Fc [138]. A fourth strategy is dedicated to improving efficacy via rational combination approaches. Simultaneous expression of up to three mAbs against multiple dengue virus serotypes, e.g., increased the breadth of viral coverage in mice [136]. Similarly, combining a DNA-based mAb with DNA-based antigen vaccination improved protection against the Chikungunya virus in mice [29].

Naked pDNA is not considered as immunogenic as viral vectors. The low immunogenicity of pDNA, however, does not exclude a humoral response against the expressed mAb. Indeed, serum mAb titers in mice have shown to decline in vivo 7–14 days after intramuscular pDNA electrotransfer due to the development of an antibody response against the expressed human mAb [132]. In contrast, the pDNA-mediated production of a fully murine mAb persisted in the circulation for over 7 months [132]. Similar findings were observed in subsequent studies, where mAb-encoding pDNA electrotransfer in immune compromised mice showed no decrease in mAb levels after a month [133].

The above studies consistently used intramuscular electrotransfer to induce mAb expression. One notable exception is the study by Kitaguchi et al. [133] in 2005, in which HTI and electrotransfer were evaluated side by side. HTI of a 40 µg pDNA dose led to single-digit µg/ml mAb peak titers in plasma 3 days after injection. mAb levels, however, rapidly decreased and detection was virtually gone after 100 days. Intramuscular electrotransfer of an identical pDNA dose led to tenfold lower peak mAb levels at day 7, with little decrease towards the end of the 100 day follow-up [133]. Despite the higher mAb peak titers, HTI has not been further pursued for pDNA-based antibody gene transfer, possibly because of its difficult translation to the clinic.

Limited data is available for pDNA-mediated antibody gene transfer other than in mice. Tjelle et al. [132] in 2004 injected sheep, weighing 15–17 kg, intramuscularly with 100 μg of pDNA-encoding murine mAb, followed by electroporation. Six of seven sheep had detectable serum mAb levels at 30–50 ng/ml, up to 2 weeks after pDNA administration. An abrupt decline in mAb levels 2 weeks after pDNA administration was linked to antibody responses raised against the foreign mouse mAb. Considering these results were obtained with a dose similar to that delivered in mice and using a non-optimal mAb sequence, there clearly remained room for improvement [132]. Recently presented data showed transient low-single-digit µg/ml serum levels of human mAbs in rabbits and NHP, following hyaluronidase pre-treatment and intramuscular electrotransfer of several milligrams of mAb-encoding pDNA [139]. Albeit promising, these mAb titers remain a far cry from those attained with viral vectors in NHP. Moreover, these animal models are still significantly smaller than a human adult, leaving the question of translatability unanswered.

In 2010, a first Phase I–II of DNA-based antibody therapy was initiated by Scancell Ltd (UK) (ClinicalTrials.gov: NCT01138410). In patients with stage 3–4 melanoma, a pDNA that encodes SCIB1, a mAb engineered to stimulate a T-cell response against melanoma, was administered intramuscularly followed by electroporation [140, 141]. Outcome measures included safety, tolerability, and biological and clinical effects. During a time period of 5.5 months, patients received five intramuscular doses of the SCIB1-encoding pDNA. The first part of the trial was a dose-escalation study (0.4, 2, 4, and 8 mg). The Clinical Study Report, released in January 2017 by the company, stated that SCIB1 was safe and well tolerated. mAb expression led to dose-dependent immunological responses and proof of clinical activity in some of the patients, with 8 mg identified as the preferential pDNA dose. mAb pharmacokinetics were not reported [142].

### Minicircle DNA

mcDNA-based antibody gene transfer is fairly recent and so far limited to studies in mice. In contrast to pDNA, mcDNA has been exclusively administered  via HTI. Yi et al. in 2014 were the first to evaluate mcDNA for antibody gene transfer, focusing on two marketed mAbs in the field of inflammatory diseases: tocilizumab, a mAb against interleukin 6 receptor, and etanercept, an Fc fusion protein against soluble TNF [143]. Fifteen days after HTI of 16 μg of mcDNA, tocilizumab peaked at 40–60 ng/ml, and dropped below 20 ng/ml 30 days after mcDNA administration. Etanercept was detected at 0.3 ng/ml 5 days after mcDNA HTI, but decreased shortly after and was no longer detected 15 days after injection. Despite the low mAb titers, a slowing incidence and improvement in arthritis symptoms were observed in mice [143]. Building on these results, the same research group constructed a fusion construct of tocilizumab and etanercept [144]. Five days following HTI of 20 μg of mcDNA that encoded tocilizumab, etanercept or the novel fusion construct, serum levels of the corresponding proteins peaked at 0.2–0.4 ng/ml and were detectable for at least 10 days after mcDNA administration. Albeit extremely low, these titers were reportedly sufficient to improve skin allograft survival [144]. In a subsequent pre-clinical study, a similar mcDNA-encoded fusion protein was successfully evaluated for arthritis [145]. In another study by the same group, abatacept, a CTLA4-Fc fusion protein approved for autoimmune diseases, was encoded in pDNA and mcDNA [146]. HTI of the abatacept-encoding mcDNA resulted in a better therapeutic response compared to pDNA in arthritis mouse models. However, it was unclear whether equimolar pDNA and mcDNA amounts were compared. Moreover, neither the concentration nor duration of the resulting abatacept expression were reported [146]. In these initial mcDNA studies [143–146], the attained serum mAb levels were up to 10,000-fold lower than reported previously for antibody-encoding pDNA HTI, despite using comparable equimolar DNA amounts [133]. The authors did not elaborate on these substantial differences. Plasma mAb titers were also lost considerably faster with mcDNA (e.g. after 15 days [143]) compared to pDNA HTI (detection for up to 100 days [133]). Although not assessed in these mcDNA studies, a humoral antibody response likely played a role, as all expressed mAb products contained human sequences. In addition to inflammatory diseases, mcDNA has been used to express a bispecific anti-CD3/CD20 mAb for the treatment of human B-cell lymphomas in a mouse cancer model [26]. Following HTI of only 5 µg of the encoding mcDNA in immune compromised mice, mAb serum levels peaked around 7 µg/ml 24 h later, and dropped sharply to 1.2 µg/ml on day 3 and to 100 ng/ml after a week. These titers were sufficient to induce an anti-lymphoma response [26] and were similar to those attained previously with higher doses of pDNA, albeit decreasing more rapidly over time [133]. Available mcDNA studies show mixed results in terms of expressed mAb titers. Indeed, the added value of mcDNA over pDNA in the context of antibody gene transfer remains subject to further research, given the lack of conclusive head-to-head comparisons.

### Recap

Compared to viral vectors, pDNA- or mcDNA-based gene transfer results in substantially lower mAb titers. Therapeutic responses in various mice models have been demonstrated (Table 1), but the lack of robust data in larger animal models currently casts doubt on its scalability and translatability to the clinic. Moving the needle on expressed mAb titers therefore remains a top priority for DNA-based antibody therapy. Various innovations, e.g. in construct engineering, show clear promise, while others, including the use of mcDNA, warrant additional evaluation. In terms of delivery, antibody-encoding DNA administration so far has been limited to i.v. (liver) and muscle (Fig. 1b). Improvements in both physical and non-physical DNA transfection methods are required to make more administration sites amendable to DNA-mediated antibody gene transfer, allowing for a broader application range.

## RNA-mediated antibody gene transfer

### Rationale

In their 1990 study, Wolff et al. [114] found that, in addition to pDNA, intramuscular injection of in vitro transcribed (IVT) mRNA also led to local expression of the encoded protein. mRNA was not pursued as actively as DNA at that time because of its low stability. Progress over the past years allowed mRNA to catch up with DNA and viral vectors as a tool for gene transfer (reviewed in [147]). Conceptually, there are several differences with these expression platforms. mRNA does not need to enter into the nucleus to be functional. Once it reaches the cytoplasm, mRNA is translated instantly. mRNA-based therapeutics are expressed more transiently compared to DNA- or viral vector-mediated gene transfer, and do not pose the risk of insertional mutagenesis in the host genome. mRNA production is relatively simple and inexpensive. In terms of administration, mRNA uptake can be enhanced using electroporation [148]. Most focus, however, has gone to non-physical transfection methods. Indeed, a variety of mRNA complexing formulations have been developed, including lipid nanoparticles (LNP), which have proven to be safe and very efficient mRNA carriers for administration in a variety of tissues and i.v. [149]. In line with this progress, IVT mRNA has reached the stage of clinical evaluation [147].

### Emerging data

Pardi et al. [150] in 2017 reported the first RNA-based antibody gene transfer study in mice using a human anti-HIV-1 mAb as model. Twenty-four hours after i.v. injection of 30 μg (1.4 mg/kg) of the encoding mRNA encapsulated in lipid nanoparticles (mRNA-LNP) in BALB/c mice, mAb plasma levels peaked at ~170 μg/ml and remained between 130 and 170 μg/ml for 5 days. mAb concentrations showed a sharp drop by day seven and were below detection 11 days post injection. In immune compromised mice, weekly i.v. injections of 30 μg (1 mg/kg) mRNA-LNP were required to maintain mAb levels above 40 μg/ml. No comparative data on the pharmacokinetics of the mAb administered as protein was included. In vivo produced mAb titers were sufficient to protect the mice from an HIV-1 challenge [150]. Already in 2008, CureVac AG (Germany) filed a patent for mRNA-based antibody gene transfer (Patent Cooperation Treaty PCT/EP2008/000081). No data was disclosed at that time. Recently presented mice data from CureVac, however, demonstrate how a single i.v. administration of mAb-encoding mRNA-LNP led to dose-dependent mAb titers, reaching low single-digit µg/ml range within 2 h after mRNA injection [44]. At the highest dose of 40 µg of mRNA-LNP, mAb expression peaked in the 10 µg/ml range after a few days. While expression lasted for at least a month, a decrease of a factor 10 or more was observed over that period of time. Of interest, this prolonged expression was also evident in immune competent mice for some of the expressed human mAbs [44]. mRNA-based expression of single-domain antibodies has also been pursued. To increase titers, serum persistence was extended via complementing single-domain antibodies with an albumin-binding peptide [44]. i.v. administration of the encoding mRNA-LNP resulted in peak levels of up to 300 µg/ml. Both i.v. and intradermal injection of mRNA-LNP encoded mAbs or single-domain antibodies resulted in therapeutic responses in mice models of infectious diseases and cancer [44] (Table 1).

### Recap

mRNA presents an emerging platform for antibody gene transfer. While the first peer-review study with mRNA-based antibodies was only recently published, this application has matured behind corporate walls for a decade or more. In addition to CureVac, Moderna Therapeutics (US) is another RNA company that is currently leveraging its platform for antibody gene transfer. Although current results differ considerably among the few available reports, mRNA may be able to rival with viral vectors in terms of generated serum mAb titers. Levels were in therapeutically relevant ranges within hours after mRNA administration, a marked shift in speed compared to DNA. The use of LNP for mRNA transfection, rather than the physical methods typically required for DNA, provide a significant advantage towards application range, if translated to the clinic. It is currently unclear how long RNA-mediated antibody expression can last. As more studies become available in the near future, the opportunities and limits of mRNA as an expression platform for antibody gene transfer will become evident.

## Remaining challenges and future directions

A broad clinical introduction of antibody gene transfer remains littered with challenges. First, it is unclear whether therapeutic mAb titers can be attained and maintained in human subjects. Second, the lack of control on mAb expression can impact safety. Third, immunogenicity against the vector or expressed mAb can limit prolonged expression. Fourth, ongoing innovations in conventional mAb therapy directly compete with antibody gene transfer, potentially impacting the relevance of the latter. For each of these challenges, possible paths forward are discussed.

### Efficacy and side-effects

The threshold for therapeutic plasma mAb titers in patients varies drastically according to the targeted disease, ranging from nanogram to tens of microgram per milliliter [22]. Despite encouraging data in pre-clinical models, it is currently uncertain whether the highlighted antibody gene transfer platforms are scalable enough to attain and maintain therapeutic mAb levels for a broad spectrum of indications in human subjects. Innovations in expression cassette, antibody format, and administration have moved the needle in each of the applied expression platforms [44, 71, 94, 137, 138, 150]. However, additional innovations remain warranted, especially in the non-viral field, to assure clinical relevance. A more local mAb production, e.g. in the tumor or brain, presents a more pragmatic way to address the possible inability of antibody gene transfer to attain systemic therapeutic levels in patients. The relative unpredictability of the expressed mAb titers presents an additional challenge. If concentrations remain below the therapeutic window for a prolonged period of time, development of resistance and inferior clinical outcomes are genuine concerns. In contrary, over-dosing may increase mAb-associated side effects, a risk that is amplified by the lack of expression control. To answer the question of pharmacokinetics, studies in more relevant animal models in terms of body mass, e.g. swine or sheep, could be pursued in anticipation of human trials. To address the current unknowns in terms of expression, clinical implementation of antibody gene transfer will likely go hand in hand with therapeutic drug monitoring.

### Control of expression

As alluded to in the previous section, a mechanism to control the duration and amount of in vivo antibody expression is a prerequisite for safe use of antibody gene transfer in many of the envisioned applications. While inducible promoters have been evaluated in mice with both viral vectored- [151] and pDNA-based antibody gene transfer [131], the applied systems are not suited for clinical translation [152]. To our knowledge, the only regulatable mechanism currently under clinical evaluation is an ecdysone-based gene switch activated by a small molecule ligand [153]. However, such an approach would require a daily drug regimen, crossing the ease of use which antibody gene transfer seeks to achieve. A more pragmatic approach to cease expression is to directly target the site of antibody gene administration, if clearly defined and contained. A transfected muscle site could e.g. be physically removed or targeted by calcium electrotransfer [154]. However, these methods are not desirable for routine use and would merely serve as an emergency-stop. Identifying a non-invasive and efficient method to permanently eliminate or tightly regulate antibody gene expression in the host therefore remains a priority. In the meantime, clinical introduction of antibody gene transfer can opt for indications where mAb expression control is considered less critical (e.g. when targeting non-self antigens in infectious diseases). For applications such as immunotherapy or inflammatory diseases, a prolonged non-controllable mAb expression presents concerns in terms of efficacy and/or side effects. The use of expression platforms (e.g. mRNA) or administration sites (e.g. tumor or skin) that may result in a more transient mAb expression can present a way forward in these indications.

### Immunogenicity and antibody characterization

Of the three expression platforms discussed, viral vectors suffer most from immunogenicity [51, 63, 87]. The development of strategies to evade pre-existing or de novo anti-vector immunity or prevent the induction of anti-vector immune responses are thus of high relevance for this field [112, 113]. In contrast, the risk of a humoral response against the expressed mAb applies to each of the expression platforms. In pre-clinical antibody gene transfer studies, the absence of an immune response has been a critical factor in achieving prolonged expression. Most, if not all, approved mAbs exhibit some level of immunogenicity when administered as conventional proteins [155]. It is currently unclear if a mAb that is in vivo expressed is more or less immunogenic than when administered as an in vitro produced protein. A risk for increased immunogenicity could occur because of the differences between natural antibody-producing plasma cells and transfected cells, e.g. muscle [87, 156–158], or because a small portion of the mAb-encoding sequence finds its way into antigen-presenting cells, where attempts to express the mAb could set off an immune response [159, 160]. The use of tissue-specific promoters or vector serotypes may be of value in this context [159]. Further work is needed to understand the factors that underlie these responses and how to circumvent them. Focus thereby should lay on the selection and design of low-immunogenic mAbs and expression platforms, rather than concomitant immunosuppressive drug regimens. Other related uncertainties are the physicochemical characteristics of in vivo expressed mAbs. Product variants (glycosylation differences, c-lysine clipped forms, etc.) and product-related impurities (truncated forms, aggregates, etc.) may vary depending on the producing cells, thereby potentially impacting mAb expression titers, efficacy and immunogenicity [161–163]. To elucidate these uncertainties, further study is needed to characterize in vivo produced mAbs.

### Positioning

Apart from antibody gene transfer, there are multiple examples of more conventional innovations that address issues with mAb therapy in terms of cost, administration and efficacy. While mAb production is anticipated to remain more expensive than e.g. small molecules or antibiotics, advancements in production technologies continue to increase yields and reduce manufacturing costs [10, 164]. Discomfort and fluctuating pharmacokinetics associated with i.v. infusion are being addressed by s.c. injection [13]. The need for frequent dosing can be overcome by extension of mAb half-life, e.g. by introducing point mutations in the mAb Fc region [165], mAb PEGylation or sustained-release formulations. The quest for more effective therapies includes the development of multispecific mAbs, which presents an alternative way to address the need for costly mAb combinations. Overall, the field for antibody gene transfer should not remain blind for these innovations, but rather take them into account when prioritizing which disease indications to go after. Relevant thereby is targeting a real unmet need and therapeutic advantage, and to focus on a rapid clinical entry by selecting the best suited expression platform. Viral-vectored antibody gene transfer in the field of HIV [95], mAb-armed oncolytic viruses, and the combination of DNA-based vaccines with DNA-based mAbs [29] all present relevant examples thereto.

## Conclusions

The state of play of antibody gene transfer is marked by substantial progress in the various interacting fields of research. While challenges persist, clinical prospects are amplified by ongoing innovations and the versatility of antibody gene transfer. In the near future, clinical introduction can be expedited by selecting the platform approach currently best suited for a mAb or disease indication of interest. Innovations in expression platform, administration and antibody technology are expected to further improve safety and efficacy, and unlock the vast clinical potential of antibody gene transfer.

## Abbreviations

AGT: antibody gene transfer
AdV: adenovirus
bnAb: broadly neutralizing antibody
CAR: chimeric antigen receptor
CNS: central nervous system
CTLA-4: cytotoxic T-lymphocyte associated protein 4
EMA: European Medicine Agency
Fab: antigen-binding fragment
Fc: fragment crystallisable
FDA: Federal Drug Administration
GM-CSF: granulocyte-macrophage colony-stimulating factor
HER2: human epidermal growth factor receptor 2
HIV: human immunodeficiency virus
HTI: hydrodynamic tail injection
IgG: immunoglobulin isotype G
i.v.: intravenous
IVT mRNA: in vitro transcribed messenger RNA
LNP: lipid nanoparticles
mAb: monoclonal antibody
mcDNA: minicircle DNA
NHP: non-human primate
PD-1: programmed cell death protein 1
PD-L1: programmed cell death-ligand 1
pDNA: plasmid DNA
rAAV: recombinant adeno-associated virus
s.c.: subcutaneous
scFv: single-chain variable fragment
SIV: simian immunodeficiency virus
VEGF: vascular endothelial growth factor

## References

[1] Ecker DM, Jones SD, Levine HL (2015). The therapeutic monoclonal antibody market. MAbs.

[2] Reichert JM (2017). Antibodies to watch in 2017. MAbs.

[3] Henricks LM, Schellens JH, Huitema AD, Beijnen JH (2015). The use of combinations of monoclonal antibodies in clinical oncology. Cancer Treat Rev.

[4] Sparrow E, Friede M, Sheikh M, Torvaldsen S (2017). Therapeutic antibodies for infectious diseases. Bull World Health Organ.

[5] Gils A, Bertolotto A, Mulleman D, Bejan-Angoulvant T, Declerck PJ (2017). Biopharmaceuticals: reference products and biosimilars to treat inflammatory diseases. Ther Drug Monit.

[6] Reichert JM. The antibody society: approved antibodies. http://www.antibodysociety.org/. Accessed 30 May 2017.

[7] Webster RM (2014). The immune checkpoint inhibitors: where are we now?. Nat Rev Drug Discov.

[8] Andrews A (2015). Treating with checkpoint inhibitors: figure $1 million per patient. Am Health Drug Benefits.

[9] Saltz LB (2016). Perspectives on cost and value in cancer care. JAMA Oncol.

[10] Kelley B (2009). Industrialization of mAb production technology: the bioprocessing industry at a crossroads. MAbs.

[11] Shaughnessy AF (2012). Monoclonal antibodies: magic bullets with a hefty price tag. BMJ.

[12] Daugherty AL, Mrsny RJ (2006). Formulation and delivery issues for monoclonal antibody therapeutics. Adv Drug Deliv Rev.

[13] Torgerson TR (2013). Overview of routes of IgG administration. J Clin Immunol.

[14] Leveque D (2014). Subcutaneous administration of anticancer agents. Anticancer Res.

[15] Beckman RA, Weiner LM, Davis HM (2007). Antibody constructs in cancer therapy: protein engineering strategies to improve exposure in solid tumors. Cancer.

[16] Dronca RS, Dong H (2015). Immunomodulatory antibody therapy of cancer: the closer, the better. Clin Cancer Res.

[17] Neves V, Aires-da-Silva F, Corte-Real S, Castanho MA (2016). Antibody approaches to treat brain diseases. Trends Biotechnol.

[18] Hodi FS, O’Day SJ, McDermott DF, Weber RW, Sosman JA, Haanen JB (2010). Improved survival with ipilimumab in patients with metastatic melanoma. N Engl J Med.

[19] Byun DJ, Wolchok JD, Rosenberg LM, Girotra M (2017). Cancer immunotherapy: immune checkpoint blockade and associated endocrinopathies. Nat Rev Endocrinol.

[20] Dumiak M (2014). Making it to manufacturing. The potential success of broadly neutralizing monoclonal antibodies for HIV prevention, treatment, and possibly even a cure could come at a cost. IAVI Rep.

[21] Sanchez-Martin D, Sanz L, Alvarez-Vallina L (2011). Engineering human cells for in vivo secretion of antibody and non-antibody therapeutic proteins. Curr Opin Biotechnol.

[22] Suscovich TJ, Alter G (2015). In situ production of therapeutic monoclonal antibodies. Expert Rev Vaccines.

[23] Johnson PR, Schnepp BC, Zhang J, Connell MJ, Greene SM, Yuste E (2009). Vector-mediated gene transfer engenders long-lived neutralizing activity and protection against SIV infection in monkeys. Nat Med.

[24] Gardner MR, Kattenhorn LM, Kondur HR, von Schaewen M, Dorfman T, Chiang JJ (2015). AAV-expressed eCD4-Ig provides durable protection from multiple SHIV challenges. Nature.

[25] DiGiandomenico A, Patel A, Smith T, Keller A, Elliot ST, Wachter L, et al. DNA-delivery of monospecific and bispecific monoclonal antibodies targeting *Pseudomonas Aeruginosa* protect mice from lethal pneumonia. Am J Respir Crit Care Med. 2016:A7898.

[26] Pang X, Ma F, Zhang P, Zhong Y, Zhang J, Wang T (2017). Treatment of human B-cell lymphomas using minicircle DNA vector expressing Anti-CD3/CD20 in a mouse model. Hum Gene Ther.

[27] Verhelle A, Nair N, Everaert I, Van Overbeke W, Supply L, Zwaenepoel O (2017). AAV9 delivered bispecific nanobody attenuates amyloid burden in the gelsolin amyloidosis mouse model. Hum Mol Genet.

[28] Muthumani K, Flingai S, Wise M, Tingey C, Ugen KE, Weiner DB (2013). Optimized and enhanced DNA plasmid vector based in vivo construction of a neutralizing anti-HIV-1 envelope glycoprotein Fab. Hum Vaccin Immunother..

[29] Muthumani K, Block P, Flingai S, Muruganantham N, Chaaithanya IK, Tingey C (2016). Rapid and long-term immunity elicited by DNA-encoded antibody prophylaxis and DNA vaccination against Chikungunya virus. J Infect Dis.

[30] Kleinpeter P, Fend L, Thioudellet C, Geist M, Sfrontato N, Koerper V (2016). Vectorization in an oncolytic vaccinia virus of an antibody, a Fab and a scFv against programmed cell death-1 (PD-1) allows their intratumoral delivery and an improved tumor-growth inhibition. Oncoimmunology..

[31] Arafat WO, Gomez-Navarro J, Buchsbaum DJ, Xiang J, Wang M, Casado E (2002). Effective single chain antibody (scFv) concentrations in vivo via adenoviral vector mediated expression of secretory scFv. Gene Ther.

[32] Afanasieva TA, Wittmer M, Vitaliti A, Ajmo M, Neri D, Klemenz R (2003). Single-chain antibody and its derivatives directed against vascular endothelial growth factor: application for antiangiogenic gene therapy. Gene Ther.

[33] Wuertzer CA, Sullivan MA, Qiu X, Federoff HJ (2008). CNS delivery of vectored prion-specific single-chain antibodies delays disease onset. Mol Ther.

[34] Zuber C, Mitteregger G, Schuhmann N, Rey C, Knackmuss S, Rupprecht W (2008). Delivery of single-chain antibodies (scFvs) directed against the 37/67 kDa laminin receptor into mice via recombinant adeno-associated viral vectors for prion disease gene therapy. J Gen Virol.

[35] Wang YJ, Pollard A, Zhong JH, Dong XY, Wu XB, Zhou HD (2009). Intramuscular delivery of a single chain antibody gene reduces brain Abeta burden in a mouse model of Alzheimer’s disease. Neurobiol Aging.

[36] Frentzen A, Yu YA, Chen N, Zhang Q, Weibel S, Raab V (2009). Anti-VEGF single-chain antibody GLAF-1 encoded by oncolytic vaccinia virus significantly enhances antitumor therapy. Proc Natl Acad Sci USA.

[37] Ryan DA, Mastrangelo MA, Narrow WC, Sullivan MA, Federoff HJ, Bowers WJ (2010). Abeta-directed single-chain antibody delivery via a serotype-1 AAV vector Improves learning behavior and pathology in Alzheimer’s disease mice. Mol Ther.

[38] Van Blarcom TJ, Sofer-Podesta C, Ang J, Boyer JL, Crystal RG, Georgiou G (2010). Affinity maturation of an anti-V antigen IgG expressed in situ through adenovirus gene delivery confers enhanced protection against Yersinia pestis challenge. Gene Ther.

[39] Weibel S, Hofmann E, Basse-Luesebrink TC, Donat U, Seubert C, Adelfinger M (2013). Treatment of malignant effusion by oncolytic virotherapy in an experimental subcutaneous xenograft model of lung cancer. J Transl Med..

[40] Patel P, Kriz J, Gravel M, Soucy G, Bareil C, Gravel C (2014). Adeno-associated virus-mediated delivery of a recombinant single-chain antibody against misfolded superoxide dismutase for treatment of amyotrophic lateral sclerosis. Mol Ther.

[41] Deshane J, Siegal GP, Alvarez RD, Wang MH, Feng M, Cabrera G (1995). Targeted tumor killing via an intracellular antibody against erbB-2. J Clin Invest..

[42] Moayeri M, Tremblay JM, Debatis M, Dmitriev IP, Kashentseva EA, Yeh AJ (2016). Adenoviral expression of a bispecific VHH-based neutralizing agent that targets protective antigen provides prophylactic protection from anthrax in mice. Clin Vaccine Immunol.

[43] Yang Z, Shi L, Yu H, Zhang Y, Chen K, Saint Fleur A (2016). Intravenous adenovirus expressing a multi-specific, single-domain antibody neutralizing TcdA and TcdB protects mice from Clostridium difficile infection. Pathog Dis..

[44] Horscroft N (2017). RNAntibody^®^—a potent mRNA technology for passive and therapeutic immunization.

[45] Mukherjee J, Dmitriev I, Debatis M, Tremblay JM, Beamer G, Kashentseva EA (2014). Prolonged prophylactic protection from botulism with a single adenovirus treatment promoting serum expression of a VHH-based antitoxin protein. PLoS ONE.

[46] Deal CE, Balazs AB (2015). Engineering humoral immunity as prophylaxis or therapy. Curr Opin Immunol.

[47] Lu QL, Bou-Gharios G, Partridge TA (2003). Non-viral gene delivery in skeletal muscle: a protein factory. Gene Ther.

[48] Ratanamart J, Shaw JA (2006). Plasmid-mediated muscle-targeted gene therapy for circulating therapeutic protein replacement: a tale of the tortoise and the hare?. Curr Gene Ther.

[49] Gene Therapy Clinical Trials Worldwide 2017. http://www.abedia.com/wiley/. Accessed 30 May 2017.

[50] Hareendran S, Balakrishnan B, Sen D, Kumar S, Srivastava A, Jayandharan GR (2013). Adeno-associated virus (AAV) vectors in gene therapy: immune challenges and strategies to circumvent them. Rev Med Virol.

[51] Baldo A, van den Akker E, Bergmans HE, Lim F, Pauwels K (2013). General considerations on the biosafety of virus-derived vectors used in gene therapy and vaccination. Curr Gene Ther.

[52] Vigna E, Pacchiana G, Mazzone M, Chiriaco C, Fontani L, Basilico C (2008). “Active” cancer immunotherapy by anti-Met antibody gene transfer. Cancer Res.

[53] Li M, Wu Y, Qiu Y, Yao Z, Liu S, Liu Y (2012). 2A peptide-based, lentivirus-mediated anti-death receptor 5 chimeric antibody expression prevents tumor growth in nude mice. Mol Ther.

[54] Wold WS, Toth K (2013). Adenovirus vectors for gene therapy, vaccination and cancer gene therapy. Curr Gene Ther.

[55] Tutykhina IL, Sedova ES, Gribova IY, Ivanova TI, Vasilev LA, Rutovskaya MV (2013). Passive immunization with a recombinant adenovirus expressing an HA (H5)-specific single-domain antibody protects mice from lethal influenza infection. Antiviral Res.

[56] Watanabe M, Boyer JL, Crystal RG (2009). Genetic delivery of bevacizumab to suppress vascular endothelial growth factor-induced high-permeability pulmonary edema. Hum Gene Ther.

[57] Skaricic D, Traube C, De B, Joh J, Boyer J, Crystal RG (2008). Genetic delivery of an anti-RSV antibody to protect against pulmonary infection with RSV. Virology.

[58] Noel D, Pelegrin M, Kramer S, Jacquet C, Skander N, Piechaczyk M (2002). High in vivo production of a model monoclonal antibody on adenoviral gene transfer. Hum Gene Ther.

[59] Jiang M, Shi W, Zhang Q, Wang X, Guo M, Cui Z (2006). Gene therapy using adenovirus-mediated full-length anti-HER-2 antibody for HER-2 overexpression cancers. Clin Cancer Res.

[60] Deshane J, Siegal GP, Wang M, Wright M, Bucy RP, Alvarez RD (1997). Transductional efficacy and safety of an intraperitoneally delivered adenovirus encoding an anti-erbB-2 intracellular single-chain antibody for ovarian cancer gene therapy. Gynecol Oncol.

[61] Alvarez RD, Curiel DT (1997). A phase I study of recombinant adenovirus vector-mediated delivery of an anti-erbB-2 single-chain (sFv) antibody gene for previously treated ovarian and extraovarian cancer patients. Hum Gene Ther.

[62] Alvarez RD, Barnes MN, Gomez-Navarro J, Wang M, Strong TV, Arafat W (2000). A cancer gene therapy approach utilizing an anti-erbB-2 single-chain antibody-encoding adenovirus (AD21): a phase I trial. Clin Cancer Res.

[63] Fausther-Bovendo H, Kobinger GP (2014). Pre-existing immunity against Ad vectors: humoral, cellular, and innate response, what’s important?. Hum Vaccin Immunother.

[64] Sibbald B (2001). Death but one unintended consequence of gene-therapy trial. CMAJ.

[65] Wu Z, Asokan A, Samulski RJ (2006). Adeno-associated virus serotypes: vector toolkit for human gene therapy. Mol Ther.

[66] Chandler RJ, Sands M, Venditti CP (2017). rAAV integration and genotoxicity: insights from animal models. Hum Gene Ther.

[67] Buchlis G, Podsakoff GM, Radu A, Hawk SM, Flake AW, Mingozzi F (2012). Factor IX expression in skeletal muscle of a severe hemophilia B patient 10 years after AAV-mediated gene transfer. Blood.

[68] Moran N (2012). First gene therapy approved. Nat Biotechnol.

[69] Morrison C. $1-million price tag set for Glybera gene therapy. Nat Biotechnol. 2015:217–8. 10.1038/nbt0315-217 25748892

[70] Lewis AD, Chen R, Montefiori DC, Johnson PR, Clark KR (2002). Generation of neutralizing activity against human immunodeficiency virus type 1 in serum by antibody gene transfer. J Virol.

[71] Fang J, Qian JJ, Yi S, Harding TC, Tu GH, VanRoey M (2005). Stable antibody expression at therapeutic levels using the 2A peptide. Nat Biotechnol.

[72] Limberis MP, Tretiakova A, Nambiar K, Wong G, Racine T, Crosariol M (2016). Adeno-associated virus serotype 9-expressed ZMapp in mice confers protection against systemic and airway-acquired ebola virus infection. J Infect Dis.

[73] Fukuchi K, Tahara K, Kim HD, Maxwell JA, Lewis TL, Accavitti-Loper MA (2006). Anti-Abeta single-chain antibody delivery via adeno-associated virus for treatment of Alzheimer’s disease. Neurobiol Dis.

[74] Sudol KL, Mastrangelo MA, Narrow WC, Frazer ME, Levites YR, Golde TE (2009). Generating differentially targeted amyloid-beta specific intrabodies as a passive vaccination strategy for Alzheimer’s disease. Mol Ther.

[75] Kou J, Kim H, Pattanayak A, Song M, Lim JE, Taguchi H (2011). Anti-amyloid-beta single-chain antibody brain delivery via AAV reduces amyloid load but may increase cerebral hemorrhages in an Alzheimer’s disease mouse model. J Alzheimers Dis.

[76] Snyder-Keller A, McLear JA, Hathorn T, Messer A (2010). Early or late-stage anti-N-terminal Huntingtin intrabody gene therapy reduces pathological features in B6.HDR6/1 mice. J Neuropathol Exp Neurol.

[77] Kou J, Yang J, Lim JE, Pattanayak A, Song M, Planque S (2015). Catalytic immunoglobulin gene delivery in a mouse model of Alzheimer’s disease: prophylactic and therapeutic applications. Mol Neurobiol.

[78] Liu W, Zhao L, Blackman B, Parmar M, Wong MY, Woo T (2016). Vectored intracerebral immunization with the anti-tau monoclonal antibody PHF1 markedly reduces tau pathology in mutant tau transgenic mice. J Neurosci.

[79] Limberis MP, Adam VS, Wong G, Gren J, Kobasa D, Ross TM (2013). Intranasal antibody gene transfer in mice and ferrets elicits broad protection against pandemic influenza. Sci Transl Med.

[80] Adam VS, Crosariol M, Kumar S, Ge MQ, Czack SE, Roy S (2014). Adeno-associated virus 9-mediated airway expression of antibody protects old and immunodeficient mice against influenza virus. Clin Vaccine Immunol.

[81] Mao Y, Kiss S, Boyer JL, Hackett NR, Qiu J, Carbone A (2011). Persistent suppression of ocular neovascularization with intravitreal administration of AAVrh. 10 coding for bevacizumab. Hum Gene Ther.

[82] De BP, Hackett NR, Crystal RG, Boyer JL (2008). Rapid/sustained anti-anthrax passive immunity mediated by co-administration of Ad/AAV. Mol Ther.

[83] Watanabe M, Boyer JL, Crystal RG (2010). AAVrh. 10-mediated genetic delivery of bevacizumab to the pleura to provide local anti-VEGF to suppress growth of metastatic lung tumors. Gene Ther.

[84] Xie Y, Hicks MJ, Kaminsky SM, Moore MA, Crystal RG, Rafii A (2014). AAV-mediated persistent bevacizumab therapy suppresses tumor growth of ovarian cancer. Gynecol Oncol.

[85] Balazs AB, West AP, Jr. Antibody gene transfer for HIV immunoprophylaxis. Nat Immunol. 2013:1–5. 10.1038/ni.2480 PMC4560170 23238748

[86] Schnepp BC, Johnson PR (2015). Vector-mediated antibody gene transfer for infectious diseases. Adv Exp Med Biol.

[87] Fuchs SP, Desrosiers RC (2016). Promise and problems associated with the use of recombinant AAV for the delivery of anti-HIV antibodies. Mol Ther Methods Clin Dev.

[88] Brady JM, Baltimore D, Balazs AB (2017). Antibody gene transfer with adeno-associated viral vectors as a method for HIV prevention. Immunol Rev.

[89] Caskey M, Klein F, Nussenzweig MC (2016). Broadly neutralizing antibodies for HIV-1 prevention or immunotherapy. N Engl J Med.

[90] Saunders KO, Wang L, Joyce MG, Yang ZY, Balazs AB, Cheng C (2015). Broadly neutralizing human immunodeficiency virus type 1 antibody gene transfer protects nonhuman primates from mucosal simian-human immunodeficiency virus infection. J Virol.

[91] Fuchs SP, Martinez-Navio JM, Piatak M, Lifson JD, Gao G, Desrosiers RC (2015). AAV-delivered antibody mediates significant protective effects against SIVmac239 challenge in the absence of neutralizing activity. PLoS Pathog.

[92] Martinez-Navio JM, Fuchs SP, Pedreno-Lopez S, Rakasz EG, Gao G, Desrosiers RC (2016). Host anti-antibody responses following adeno-associated virus-mediated delivery of antibodies against HIV and SIV in Rhesus monkeys. Mol Ther.

[93] Schnepp BC, Johnson PR (2014). Adeno-associated virus delivery of broadly neutralizing antibodies. Curr Opin HIV AIDS..

[94] Fuchs SP, Martinez-Navio JM, Gao G, Desrosiers RC (2016). Recombinant AAV vectors for enhanced expression of authentic IgG. PLoS ONE.

[95] HIV immunity goes direct. Nat Biotechnol. 2014:397. 10.1038/nbt.2907 24811492

[96] Kaufman HL, Kohlhapp FJ, Zloza A (2015). Oncolytic viruses: a new class of immunotherapy drugs. Nat Rev Drug Discov.

[97] Ledford H (2015). Cancer-fighting viruses win approval. Nature.

[98] Liu TC, Thorne SH, Kirn DH (2008). Oncolytic adenoviruses for cancer gene therapy. Methods Mol Biol.

[99] Gholami S, Marano A, Chen NG, Aguilar RJ, Frentzen A, Chen CH (2014). A novel vaccinia virus with dual oncolytic and anti-angiogenic therapeutic effects against triple-negative breast cancer. Breast Cancer Res Treat.

[100] Buckel L, Advani SJ, Frentzen A, Zhang Q, Yu YA, Chen NG (2013). Combination of fractionated irradiation with anti-VEGF expressing vaccinia virus therapy enhances tumor control by simultaneous radiosensitization of tumor associated endothelium. Int J Cancer.

[101] Adelfinger M, Bessler S, Frentzen A, Cecil A, Langbein-Laugwitz J, Gentschev I (2015). Preclinical testing oncolytic vaccinia virus strain GLV-5b451 expressing an anti-VEGF single-chain antibody for canine cancer therapy. Viruses.

[102] Patil SS, Gentschev I, Adelfinger M, Donat U, Hess M, Weibel S (2012). Virotherapy of canine tumors with oncolytic vaccinia virus GLV-1h109 expressing an anti-VEGF single-chain antibody. PLoS ONE.

[103] Huang T, Wang H, Chen NG, Frentzen A, Minev B, Szalay AA (2015). Expression of anti-VEGF antibody together with anti-EGFR or anti-FAP enhances tumor regression as a result of vaccinia virotherapy. Mol Ther Oncolytics..

[104] Liikanen I, Tahtinen S, Guse K, Gutmann T, Savola P, Oksanen M (2016). Oncolytic adenovirus expressing monoclonal antibody trastuzumab for treatment of HER2-positive cancer. Mol Cancer Ther.

[105] Lichty BD, Breitbach CJ, Stojdl DF, Bell JC (2014). Going viral with cancer immunotherapy. Nat Rev Cancer.

[106] Dias JD, Hemminki O, Diaconu I, Hirvinen M, Bonetti A, Guse K (2012). Targeted cancer immunotherapy with oncolytic adenovirus coding for a fully human monoclonal antibody specific for CTLA-4. Gene Ther.

[107] Du T, Shi G, Li YM, Zhang JF, Tian HW, Wei YQ (2014). Tumor-specific oncolytic adenoviruses expressing granulocyte macrophage colony-stimulating factor or anti-CTLA4 antibody for the treatment of cancers. Cancer Gene Ther.

[108] Engeland CE, Grossardt C, Veinalde R, Bossow S, Lutz D, Kaufmann JK (2014). CTLA-4 and PD-L1 checkpoint blockade enhances oncolytic measles virus therapy. Mol Ther.

[109] Tanoue K, Rosewell Shaw A, Watanabe N, Porter CE, Rana B, Gottschalk S (2017). Armed oncolytic adenovirus expressing PD-L1 mini-body enhances anti-tumor effects of chimeric antigen receptor T-cells in solid tumors. Cancer Res.

[110] Louis Jeune V, Joergensen JA, Hajjar RJ, Weber T (2013). Pre-existing anti-adeno-associated virus antibodies as a challenge in AAV gene therapy. Hum Gene Ther Methods.

[111] Ratanamart J, Huggins CG, Shaw JA (2010). Transgene expression in mononuclear muscle cells not infiltrating inflammatory cells following intramuscular plasmid gene electrotransfer. J Gene Med..

[112] Tseng YS, Agbandje-McKenna M (2014). Mapping the AAV capsid host antibody response toward the development of second generation gene delivery vectors. Front Immunol.

[113] Basner-Tschakarjan E, Mingozzi F (2014). Cell-mediated immunity to AAV vectors, evolving concepts and potential solutions. Front Immunol..

[114] Wolff JA, Malone RW, Williams P, Chong W, Acsadi G, Jani A (1990). Direct gene transfer into mouse muscle in vivo. Science.

[115] Hardee CL, Arevalo-Soliz LM, Hornstein BD, Zechiedrich L (2017). Advances in non-viral DNA vectors for gene therapy. Genes.

[116] Faurez F, Dory D, Le Moigne V, Gravier R, Jestin A (2010). Biosafety of DNA vaccines: new generation of DNA vectors and current knowledge on the fate of plasmids after injection. Vaccine.

[117] Heller R, Heller LC (2015). Gene electrotransfer clinical trials. Adv Genet.

[118] Daud AI, DeConti RC, Andrews S, Urbas P, Riker AI, Sondak VK (2008). Phase I trial of interleukin-12 plasmid electroporation in patients with metastatic melanoma. J Clin Oncol.

[119] Evans C, Hannaman D, Singh Manmohan (2013). Current status of electroporation technologies for vaccine delivery. Novel immune potentiators and delivery technologies for next generation vaccines.

[120] Diehl MC, Lee JC, Daniels SE, Tebas P, Khan AS, Giffear M (2013). Tolerability of intramuscular and intradermal delivery by CELLECTRA adaptive constant current electroporation device in healthy volunteers. Hum Vaccin Immunother.

[121] Suda T, Liu D (2007). Hydrodynamic gene delivery: its principles and applications. Mol Ther.

[122] Meacham JM, Durvasula K, Degertekin FL, Fedorov AG (2014). Physical methods for intracellular delivery: practical aspects from laboratory use to industrial-scale processing. J Lab Autom.

[123] Ibraheem D, Elaissari A, Fessi H (2014). Gene therapy and DNA delivery systems. Int J Pharm.

[124] Simcikova M, Prather KL, Prazeres DM, Monteiro GA (2015). Towards effective non-viral gene delivery vector. Biotechnol Genet Eng Rev.

[125] Vandermeulen G, Marie C, Scherman D, Préat V (2011). New generation of plasmid backbones devoid of antibiotic resistance marker for gene therapy trials. Mol Ther.

[126] Takahashi Y, Nishikawa M, Takakura Y (2012). Development of safe and effective nonviral gene therapy by eliminating CpG motifs from plasmid DNA vector. Front Biosci.

[127] Chen ZY, He CY, Meuse L, Kay MA (2004). Silencing of episomal transgene expression by plasmid bacterial DNA elements in vivo. Gene Ther.

[128] Mayrhofer P, Schleef M, Jechlinger W, Wolfgang W, Ulrike SS (2009). Use of minicircle plasmids for gene therapy. Methods in molecular biology, gene therapy of cancer.

[129] Chen ZY, He CY, Ehrhardt A, Kay MA (2003). Minicircle DNA vectors devoid of bacterial DNA result in persistent and high-level transgene expression in vivo. Mol Ther.

[130] Kobelt D, Schleef M, Schmeer M, Aumann J, Schlag PM, Walther W (2013). Performance of high quality minicircle DNA for in vitro and in vivo gene transfer. Mol Biotechnol.

[131] Perez N, Bigey P, Scherman D, Danos O, Piechaczyk M, Pelegrin M (2004). Regulatable systemic production of monoclonal antibodies by in vivo muscle electroporation. Genet Vaccines Ther..

[132] Tjelle TE, Corthay A, Lunde E, Sandlie I, Michaelsen TE, Mathiesen I (2004). Monoclonal antibodies produced by muscle after plasmid injection and electroporation. Mol Ther.

[133] Kitaguchi K, Toda M, Takekoshi M, Maeda F, Muramatsu T, Murai A (2005). Immune deficiency enhances expression of recombinant human antibody in mice after nonviral in vivo gene transfer. Int J Mol Med.

[134] Yamazaki T, Nagashima M, Ninomiya D, Arai Y, Teshima Y, Fujimoto A (2011). Passive immune-prophylaxis against influenza virus infection by the expression of neutralizing anti-hemagglutinin monoclonal antibodies from plasmids. Jpn J Infect Dis..

[135] McMahon JM, Signori E, Wells KE, Fazio VM, Wells DJ (2001). Optimisation of electrotransfer of plasmid into skeletal muscle by pretreatment with hyaluronidase: increased expression with reduced muscle damage. Gene Ther.

[136] Flingai S, Plummer EM, Patel A, Shresta S, Mendoza JM, Broderick KE (2015). Protection against dengue disease by synthetic nucleic acid antibody prophylaxis/immunotherapy. Sci Rep..

[137] Cooch N, Guibinga G, Chen J, Reed C, Ramos S, Smith T, et al. Description of structural modifications of DNA vector encoded monoclonal antibodies (DMAbs) to improve in vivo expression levels after intramuscular injection and electroporation. American Society for Gene and Cell Therapy Annual Meeting; Washington, DC, May 9–14, 2017.

[138] Guibinga GH, Chen J, Reed C, Cooch N, Ramos S, Yan J, Smith T, Patel A, et al. Functional assessment of structural reformatting and protein engineering strategies for therapeutic gene transfer synthetic DNA-plasmid encoding antibodies against ebola virus disease (EVD). American Society for Gene and Cell Therapy Annual Meeting; Washington DC, May 9–14, 2017.

[139] Schultheis K, Smith TRF, Ramos S, Schommer N, Jian J, Yung B, et al. Optimization of gene transfer protocol achieves robust in vivo expression of DNA-based monoclonal antibodies (DMAbs) in small and large animals. American Society for Gene and Cell Therapy Annual Meeting; Washington DC, May 9–14, 2017.

[140] Goodfellow R (2015). Scancell’s vaccine SCIB1 could help to prevent recurrence of melanoma. Immunotherapy.

[141] Xue W, Brentville VA, Symonds P, Cook KW, Yagita H, Metheringham RL (2016). SCIB1, a huIgG1 antibody DNA vaccination, combined with PD-1 blockade induced efficient therapy of poorly immunogenic tumors. Oncotarget.

[142] Scancell Limited. SCIB1 clinical study report 2017. http://www.scancell.co.uk/news/regulatory-news/scib1-clinical-study-report. Accessed 30 May 2017.

[143] Yi H, Kim Y, Kim J, Jung H, Rim YA, Jung SM (2014). A new strategy to deliver synthetic protein drugs: self-reproducible biologics using minicircles. Sci Rep.

[144] Lim SW, Kim YK, Park N, Jin L, Jin J, Doh KC (2015). Application of minicircle technology of self-reproducing synthetic protein drugs in preventing skin allograft rejection. Ann Transpl.

[145] Kim Y, Yi H, Jung H, Rim YA, Park N, Kim J (2016). A dual target-directed agent against interleukin-6 receptor and tumor necrosis factor alpha ameliorates experimental arthritis. Sci Rep..

[146] Rim YA, Yi H, Kim Y, Park N, Jung H, Kim J (2014). Self in vivo production of a synthetic biological drug CTLA4Ig using a minicircle vector. Sci Rep.

[147] Sahin U, Kariko K, Tureci O (2014). mRNA-based therapeutics: developing a new class of drugs. Nat Rev Drug Discov.

[148] Broderick KE, Humeau LM (2017). Enhanced delivery of DNA or RNA vaccines by electroporation. Methods Mol Biol.

[149] Pardi N, Tuyishime S, Muramatsu H, Kariko K, Mui BL, Tam YK (2015). Expression kinetics of nucleoside-modified mRNA delivered in lipid nanoparticles to mice by various routes. J Control Release.

[150] Pardi N, Secreto AJ, Shan X, Debonera F, Glover J, Yi Y (2017). Administration of nucleoside-modified mRNA encoding broadly neutralizing antibody protects humanized mice from HIV-1 challenge. Nat Commun.

[151] Fang J, Yi S, Simmons A, Tu GH, Nguyen M, Harding TC (2007). An antibody delivery system for regulated expression of therapeutic levels of monoclonal antibodies in vivo. Mol Ther.

[152] Guo ZS, Li Q, Bartlett DL, Yang JY, Fang B (2008). Gene transfer: the challenge of regulated gene expression. Trends Mol Med..

[153] Cai H, Sun L, Miao J, Krishman S, Lebel F, Barrett JA (2016). Plasma pharmacokinetics of veledimex, a small-molecule activator ligand for a proprietary gene therapy promoter system, in healthy subjects. Clin Pharmacol Drug Dev.

[154] Hojman P, Spanggaard I, Olsen CH, Gehl J, Gissel H (2011). Calcium electrotransfer for termination of transgene expression in muscle. Hum Gene Ther.

[155] Schellekens H (2010). The immunogenicity of therapeutic proteins. Discov Med..

[156] Cao O, Hoffman BE, Moghimi B, Nayak S, Cooper M, Zhou S (2009). Impact of the underlying mutation and the route of vector administration on immune responses to factor IX in gene therapy for hemophilia B. Mol Ther.

[157] Boisgerault F, Mingozzi F (2015). The skeletal muscle environment and its role in immunity and tolerance to AAV vector-mediated gene transfer. Curr Gene Ther.

[158] Shimizu Y, Meunier L, Hendershot LM (2009). pERp1 is significantly up-regulated during plasma cell differentiation and contributes to the oxidative folding of immunoglobulin. Proc Natl Acad Sci USA.

[159] Weeratna RD, Wu T, Efler SM, Zhang L, Davis HL (2001). Designing gene therapy vectors: avoiding immune responses by using tissue-specific promoters. Gene Ther.

[160] Martinez-Navio JM, Fuchs SP, Pedreno-Lopez S, Rakasz EG, Gao G, Desrosiers RC (2016). Host anti-antibody responses following AAV-mediated delivery of antibodies against HIV and SIV in Rhesus monkeys. Mol Ther.

[161] Ho SC, Koh EY, van Beers M, Mueller M, Wan C, Teo G (2013). Control of IgG LC:HC ratio in stably transfected CHO cells and study of the impact on expression, aggregation, glycosylation and conformational stability. J Biotechnol.

[162] Davies SL, O’Callaghan PM, McLeod J, Pybus LP, Sung YH, Rance J (2011). Impact of gene vector design on the control of recombinant monoclonal antibody production by Chinese hamster ovary cells. Biotechnol Prog.

[163] Jefferis R, Endrenyi L, Declerck P, Chow S-C (2017). Characterization of biosimilar biologics: the link between structure and functions. Biosimilar drug product development.

[164] Elgundi Z, Reslan M, Cruz E, Sifniotis V, Kayser V (2016). The state-of-play and future of antibody therapeutics. Adv Drug Deliv Rev.

[165] Saxena A, Wu D (2016). Advances in therapeutic Fc engineering—modulation of IgG-associated effector functions and serum half-life. Front Immunol.

[166] Sofer-Podesta C, Ang J, Hackett NR, Senina S, Perlin D, Crystal RG (2009). Adenovirus-mediated delivery of an anti-V antigen monoclonal antibody protects mice against a lethal Yersinia pestis challenge. Infect Immun.

[167] Pereboev A, Borisevich V, Tsuladze G, Shakhmatov M, Hudman D, Kazachinskaia E (2008). Genetically delivered antibody protects against West Nile virus. Antiviral Res.

[168] Han T, Abdel-Motal UM, Chang DK, Sui J, Muvaffak A, Campbell J (2012). Human anti-CCR4 minibody gene transfer for the treatment of cutaneous T-cell lymphoma. PLoS ONE.

[169] Lv F, Qiu Y, Zhang Y, Liu S, Shi J, Liu Y (2011). Adeno-associated virus-mediated anti-DR5 chimeric antibody expression suppresses human tumor growth in nude mice. Cancer Lett.

[170] Shi J, Liu Y, Zheng Y, Guo Y, Zhang J, Cheung PT (2006). Therapeutic expression of an anti-death receptor 5 single-chain fixed-variable region prevents tumor growth in mice. Cancer Res.

[171] Ho DT, Wykoff-Clary S, Gross CS, Schneider D, Jin F, Kretschmer PJ (2009). Growth inhibition of an established A431 xenograft tumor by a full-length anti-EGFR antibody following gene delivery by AAV. Cancer Gene Ther.

[172] Watanabe M, Boyer JL, Hackett NR, Qiu J, Crystal RG (2008). Genetic delivery of the murine equivalent of bevacizumab (avastin), an anti-vascular endothelial growth factor monoclonal antibody, to suppress growth of human tumors in immunodeficient mice. Hum Gene Ther.

[173] Southwell AL, Ko J, Patterson PH (2009). Intrabody gene therapy ameliorates motor, cognitive, and neuropathological symptoms in multiple mouse models of Huntington’s disease. J Neurosci.

[174] Yang J, Pattanayak A, Song M, Kou J, Taguchi H, Paul S (2013). Muscle-directed anti-Abeta single-chain antibody delivery via AAV1 reduces cerebral Abeta load in an Alzheimer’s disease mouse model. J Mol Neurosci.

[175] Moda F, Vimercati C, Campagnani I, Ruggerone M, Giaccone G, Morbin M (2012). Brain delivery of AAV9 expressing an anti-PrP monovalent antibody delays prion disease in mice. Prion..

[176] de Jong YP, Dorner M, Mommersteeg MC, Xiao JW, Balazs AB, Robbins JB (2014). Broadly neutralizing antibodies abrogate established hepatitis C virus infection. Sci Transl Med.

[177] Balazs AB, Chen J, Hong CM, Rao DS, Yang L, Baltimore D (2012). Antibody-based protection against HIV infection by vectored immunoprophylaxis. Nature.

[178] Balazs AB, Ouyang Y, Hong CM, Chen J, Nguyen SM, Rao DS (2014). Vectored immunoprophylaxis protects humanized mice from mucosal HIV transmission. Nat Med.

[179] Horwitz JA, Halper-Stromberg A, Mouquet H, Gitlin AD, Tretiakova A, Eisenreich TR (2013). HIV-1 suppression and durable control by combining single broadly neutralizing antibodies and antiretroviral drugs in humanized mice. Proc Natl Acad Sci USA.

[180] Balazs AB, Bloom JD, Hong CM, Rao DS, Baltimore D (2013). Broad protection against influenza infection by vectored immunoprophylaxis in mice. Nat Biotechnol.

[181] Deal C, Balazs AB, Espinosa DA, Zavala F, Baltimore D, Ketner G (2014). Vectored antibody gene delivery protects against Plasmodium falciparum sporozoite challenge in mice. Proc Natl Acad Sci USA.

[182] Hicks MJ, Rosenberg JB, De BP, Pagovich OE, Young CN, Qiu JP (2012). AAV-directed persistent expression of a gene encoding anti-nicotine antibody for smoking cessation. Sci Transl Med..

[183] Rosenberg JB, Hicks MJ, De BP, Pagovich O, Frenk E, Janda KD (2012). AAVrh. 10-mediated expression of an anti-cocaine antibody mediates persistent passive immunization that suppresses cocaine-induced behavior. Hum Gene Ther.

[184] Li J, Olvera AI, Akbari OS, Moradian A, Sweredoski MJ, Hess S (2015). Vectored antibody gene delivery mediates long-term contraception. Curr Biol.

[185] Hollevoet K, Geukens N, Velde GV, Declerck P (2015). Long-term in vivo expression of trastuzumab following intramuscular electrotransfer of the encoding DNA in mice. J Immunother Cancer.

[186] Muthumani K, Chung C, Agarwal S, Plyler R, Kudchodkar S, Flingai S (2016). In vivo expression of plasmid encoded IgG for PD-1 or LAG3 by synthetic DNA as a new tool for cancer immunotherapy. Mol Ther.

[187] Patel A, Davis C, Park DH, Smith TRF, Leung A, Tierney K, et al. DNA-monoclonal antibody gene delivery against ebola virus disease, an in vivo DNA vectored approach for achieving sustained, transient serum levels of protective IgG. American Society for Gene and Cell Therapy Annual Meeting; Washington DC, May 9–14, 2017.

